# Safety of ribavirin in cockatiels (*Nymphicus hollandicus)* – a preliminary study

**DOI:** 10.1038/s41598-025-22268-9

**Published:** 2025-11-03

**Authors:** Ines Szotowska, Aleksandra Ledwoń, Michał Czopowicz

**Affiliations:** 1 Department of Pathology and Veterinary Diagnostics, Warsaw University of Life Sciences, Nowoursynowska 159c, Warsaw, 02-776 Poland; 2 Division of Veterinary Epidemiology and Economics, Institute of Veterinary Medicine, Warsaw University of Life Sciences, Nowoursynowska 159c, Warsaw, 02-776 Poland

**Keywords:** Ribavirin, Cockatiel, Antiviral drugs, Avian viral diseases, Antiviral therapy, Diseases, Drug discovery, Medical research

## Abstract

Viral infections remain a major health concern in psittacine birds, with avian bornaviruses (ABV) causing proventricular dilatation disease (PDD), a chronic and often fatal condition. Ribavirin, a broad-spectrum antiviral drug widely used in human medicine, has shown in vitro (ABV) and *in ovo* (Newcastle disease virus) efficacy against some avian viruses, but its safety profile in birds is largely unknown. This study aimed to evaluate the safety of prolonged ribavirin administration in healthy cockatiels (*Nymphicus hollandicus*) aged approximately 6 months. The experiment consisted of experimental group (10 cockatiels) and negative control group (10 cockatiels). Ribavirin was administered to the experimental cockatiels according to the following protocol: orally (by gavage at dose 30 mg/kg body weight [BW]/day) two consecutive 28-day courses (course 1 & 2) separated and followed by 14-day recovery periods, then 15 mg/kg BW/day orally and 15 mg/kg BW/day intranasally for 28 days (course 3). The cockatiels were regularly weighed, clinically examined, and blood was collected for hematological and biochemical analyses. No clinical signs of adverse reactions were observed, neither during the therapy nor for 12 weeks after the therapy. BW changed significantly during the experiment, however without any link to ribavirin treatment. Only hematocrit decreased significantly after the ribavirin course 1. Our results indicate that ribavirin at 30 mg/kg BW, administered orally or in combination with intranasal dosing does not appear to cause apparent clinical or laboratory adverse reactions in cockatiels, except for potential alteration of hematocrit which is, however, too mild to be clinically meaningful. This provides an essential first step toward evaluating ribavirin as a therapeutic option for avian viral diseases.

**Supplementary Information:**

The online version contains supplementary material available at 10.1038/s41598-025-22268-9.

## Introduction

Viral diseases are a constant problem in companion birds, especially parrots^1^. Psittacine birds are commonly infected with beak and feather disease virus, avian polyomavirus which are DNA viruses^2,3^ and avian bornaviruses (ABV) which are RNA viruses^4,5^. Infections with these three viruses can be asymptomatic for a long time what often results in delayed diagnosis and increases risk of the spread of infection in a flock^2–5^. ABV, discovered in 2008, are currently intensively studied especially with respect to most effective treatment and prevention^5^. There is evidence that they cause the disease not only in parrots^6,7^ but also in canaries (*Serinus canaria*)^8,9^. ABV are an etiological agent of proventricular dilatation disease (PDD) also called avian ganglioneuritis. PDD is usually a chronic and fatal disease and poses a serious threat to endangered species conservation programs what has been shown in the case of Spix’s macaw (*Cyanopsitta spixii*)^10^. It is also a common cause of losses in psittacine collections and parrots kept as companion animals. It is worth to mention that beyond impact of infection diseases on avian health, pet birds also pose zoonotic risks through bacterial and viral infections, highlighting the importance of a One Health approach to prevention and management^11^. Solution to some problems associated with parrots viral diseases could be an antiviral drug - ribavirin.

Ribavirin is a purine analog with a broad-spectrum antiviral activity and has been considered as potentially effective treatment in PDD^12–14^. It has been described that ribavirin can potentially act on numerous stages of the virus life cycle such as the inhibition of translation, the inhibition of genome or transcript capping, the inhibition of RNA synthesis, causing increased mutation and production of non-viable genomes, enhancement of the antiviral immune response, and the prevention of spread and pathogenesis^15^. In human medicine, ribavirin is used mainly to treat hepatitis C virus infections^16^.

In avian medicine ribavirin is not currently used. However, in vitro and *in ovo* efficacy against some viruses which cause diseases in birds such as Newcastle disease virus (NDV) (*in ovo*)^12^ and ABV in parrots (in vitro)^17^ has been proven. Synergistic antiviral effect of ribavirin and recombinant interferon α (IFN-α) against ABV in parrots has also been shown in avian cells^13^.

Moreover, current knowledge about ribavirin toxicity in avian species is scarce. In previously mentioned study when ribavirin has been used *in ovo* also its toxicity has been assessed^12^. Inoculation of a 0.1 mL solution with the highest used concentration of 40 µg/mL was toxic, and embryos in eggs died but with lower concentrations (10 µg/mL and 20 µg/mL) toxic effect was not observed.

In rats, monkeys and human the major adverse effect of ribavirin treatment is haemolytic anemia that results from the accumulation of ribavirin’s active metabolite, ribavirin 5’-triphosphate, in red blood cells (RBCs)^18^ which can lead to RBC destruction^19,20^. In one study on rats, significant alterations of some blood chemistry parameters was also observed after 28 days of treatment with dose 120 mg/kg/day – a decrease of alkaline phosphatase (ALP) activity, cholesterol concentration, and an increase of total protein and albumin concentration^21^. There is no data about safety of ribavirin treatment in avian species including cockatiels. It could be expected that haemolytic anemia, the major adverse effect described in mammals, would be also the problem in birds.

Therefore, the aim of this study was to systematically evaluate the safety of ribavirin administered at dose of 30 mg/kg BW/day orally or in combination with intranasal route in clinically healthy cockatiels as a psittacine model, with particular emphasis on clinical condition, body weight, and hematological and biochemical parameters.

## Materials and methods

### Ethics declaration

The experiment was approved by the 2nd Local Ethics Committee for Animal Experiments in Warsaw, Poland (approval no. WAW2/002/2023). The authors confirm that they complied with the ARRIVE guidelines and all experiments were performed in accordance with the Directive 2010/63/EU of the European Parliament and of the Council of 22 September 2010 on the protection of animals used for scientific purposes and the National Animal Protection Act.

### Parrots

Twenty cockatiels (*Nymphicus hollandicus*), 11 males and 9 females, aged approximately 6 months were obtained from a breeding facility in Poland. They were clinically healthy and free from parasites and ABV infection, as confirmed by several wet mount preparation of fresh faecal samples and crop swabs and reverse transcription polymerase chain reaction (RT-PCR) respectively. The birds were housed as the only inhabitants of the aviary room (dimensions of 14 × 5 × 3 m) in cages which measured at least 79 × 48 × 78 cm, two individuals per a cage. The temperature of 21–25 °C and 12 h/12 h light/dark cycle were maintained. The cockatiels were fed *at libitum* on a standard seed diet (complete mix for parakeets), egg food, mineral supplements (grit, cuttlefish bone), fresh greens, vegetables and fruits, as well as water (two waterers and a pool in every cage). Environmental enrichment consisted of perches of various diameter and surface as well as safe hanging toys. The cockatiels underwent a 2-month quarantine before the study began.

### Design of experiment, treatment and clinical monitoring

The cockatiels were randomly assigned (simple randomization using the envelope method) to the experimental (ribavirin) and control (placebo) group. Each group counted 10 individuals – 5 females and 5 males in the experimental group and 4 females and 6 males in the control group. Distribution of sexes did not differ significantly between groups (*p* = 0.999). Group size was determined so that it allowed for detection of significant differences between groups with the power of at least 85% (β < 0.15) assuming that the standardized mean difference of numerical measurements between groups was at least 1.5 and the significance level (α) = 0.05.

Ribavirin powder ≥ 98% (Thermo Fisher Scientific Inc., Waltham, Massachusetts, USA) was dissolved in the water for injection (Polpharma, Starogard Gdański, Poland) to the final concentration of 10 mg/ml for oral administration and 30 mg/ml for intranasal administration. Fresh solutions were prepared daily. The same volume of water for injection was administered to cockatiels from the control group as a placebo. Ribavirin and placebo were administered to the cockatiels once daily at the same time. The experiment was divided into three 28-day courses (course 1, 2, and 3) of ribavirin treatment separated by two 14-day recovery periods. In the course 1 and 2, ribavirin solution was administered orally by crop gavage (16G feeding tube) at a dose of 30 mg/kg of body weight (BW)/day (0,3 ml/100 g BW/day). In the course 3, ribavirin was administered half-by-half orally by crop gavage (15 mg/kg BW) and intranasally using a laboratory pipette 15 mg/kg BW. The ribavirin course 3 was followed by the 12-week recovery period.

Cockatiels were observed once daily for at least 20 min. for the entire experiment including the 2-month quarantine and the recovery periods for clinical signs of adverse reactions. BW was measured once a week, before the morning feeding, using scale.

### Blood sample collection and testing

Blood was collected from each cockatiel 8 times: on the 1 st day of each course, on the 1 st day of each recovery period, and then 4 and 12 weeks after completion of therapy.

A sample of 0.6–1.0.6.0 ml of blood was collected every time from the right jugular vein using 25G needle attached to 1 ml syringe. Immediately after collection, blood samples were transferred into Microvette^®^ 500 Lithium heparin LH 500 µl (Sarstedt, Nümbrecht, Germany) for the complete blood count (CBC) and blood chemistry. Total leukocyte count (WBC) and differential leukocyte count (including heterophil, lymphocyte, monocyte, eosinophil, and basophil counts) was determined using a Neubauer chamber (Assistent, Germany) and a light microscope Olympus BX41 (Olympus, Tokyo, Japan). Red blood cell parameters were determined using instrumental analysis: hemoglobin concentration (HGB) – HemoCue Hb 201 (HemoCue AB, Sweden), hematocrit (Ht) – hematocrit centrifuge, erythrocyte count (RBC) – Sysmex XN-1000 V (Sysmex, USA). Blood chemistry was performed using automatic analyzer (Roche Cobas 6000, Roche, USA) and included activities of 6 enzymes: amylase (AMY), alkaline phosphatase (ALP), aspartate aminotransferase (AST), creatine kinase (CK), gamma-glutamyltransferase (GGT), and lactate dehydrogenase (LDH) as well as concentrations of 5 chemical compounds: total cholesterol, total protein, bile acids, uric acid, triglycerides, and 4 electrolytes: natrium (Na), potassium (K), calcium (Ca), and phosphate (P).

Missing values of hematological and biochemical analysis were replaced by the arithmetic mean of other measurements of the same parrot and on their basis Ht, MCV, MCH and MCHC were calculated as follows: Ht = HGB/MCHC, MCV = Ht/RBC, MCH = HGB/Ht, MCHC= (HGB/Ht)×100. Only missing values of differential leukogram were replaced using last observation carried forward (LOCF) method so that they added up to 100%.

### Statistical analysis

Normality of distribution of numerical (quantitative) variables was evaluated using the normal probability Q-Q plots and Shapiro-Wilk test. If normality assumption held, numerical variables were summarized using the arithmetic mean with 95% confidence intervals (CI 95%) and standard deviation (SD), otherwise the median and interquartile range (IQR) were used. Range and individual measurements were also presented in tables and figures, respectively. Extreme values were identified using Tukey’s rule and an observation was identified as extreme if its value was < (1st Quartile – 3×IQR) or > (3rd Quartile + 3×IQR). Repeated measure analysis of variance (RM-ANOVA) was applied to analyze the change of blood measurements between timepoints (within-subject analysis) and to compare blood measurements between experimental and control group (between-subject analysis). The RM-ANOVA was performed on raw data if the variable was considered as normally distributed, otherwise on rank-transformed data according to RM1 transformation^22^. Sphericity assumption was tested using the Mauchly’s test and if significant the epsilon for correction of degrees of freedom (df) of F-statistic was estimated according to the Greenhouse-Geisser procedure. If groups turned out to differ significantly, the Tukey’s post-hoc test for equal groups was used to identify timepoints at which the difference between groups was significant. As at some timepoints normality assumption was seriously violated in almost all blood measurements, only body weight was analyzed without a prior rank-transformation. Categorical variables were summarized as counts and proportions (%) and compared between groups using the Pearson χ^2^ test or Fisher exact test. All tests were two-tailed and α was set at 0.05. The analysis was carried out in TIBCO Statistica 13.3 (TIBCO Software Inc., Palo Alto, CA).

## Results

**Fig. 1:**
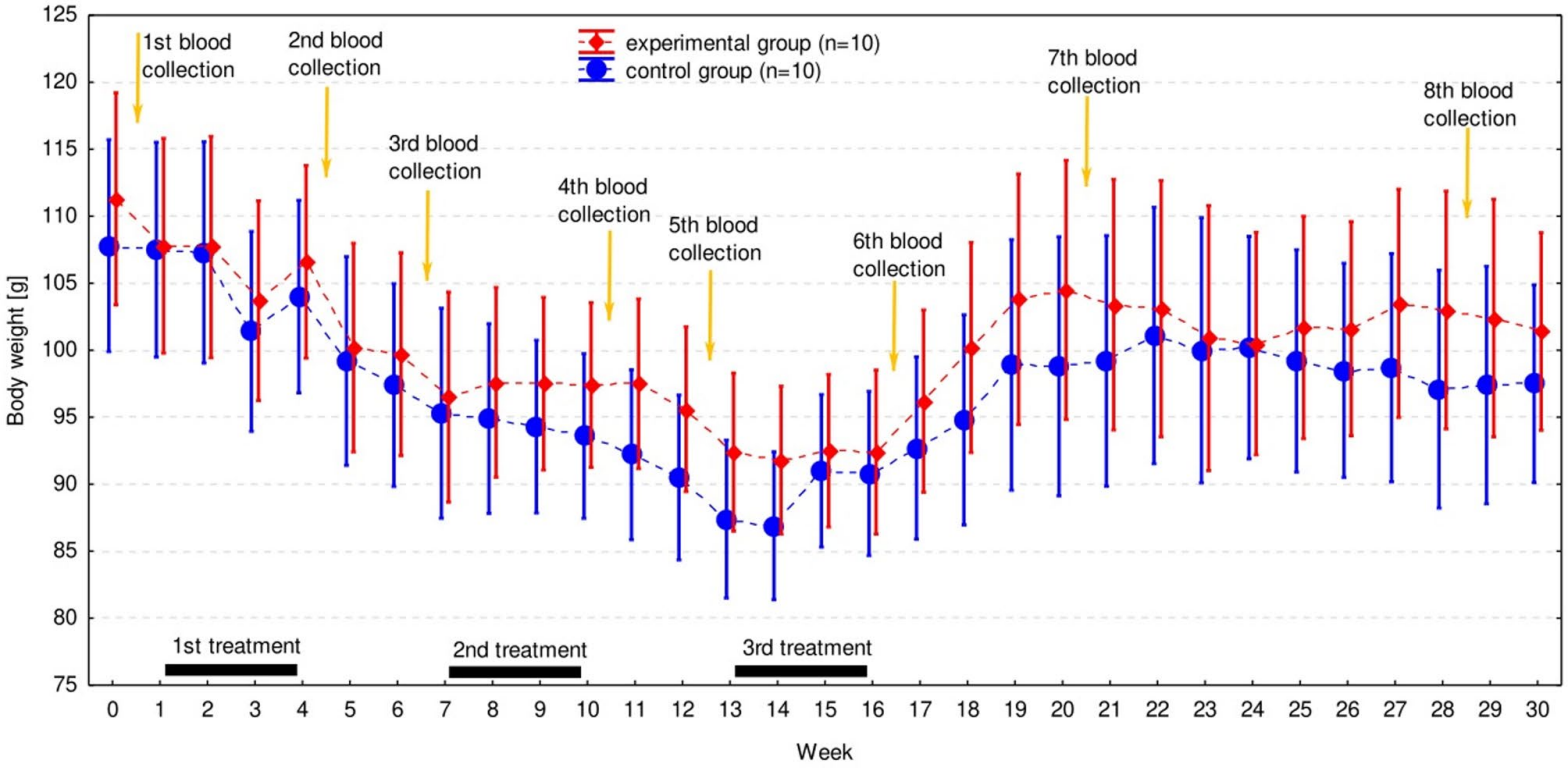
Change of body weight (BW) during the experiment in the experimental and control group. It has been presented as the arithmetic mean (solid dot or diamond) and 95% confidence intervals (whiskers). The 4-week periods of treatment are indicated by black bars. Orange arrows indicate the time of blood collection for hematology and biochemistry analysis.

No clinical signs were observed in any cockatiel of either group. BW changed significantly with time (F_1,30_ = 21.1, *p* < 0.001) but there was no regular pattern of the changes and they were similar in both groups (F_1,30_ = 0.65, *p* = 0.928). As a result no significant difference in BW was observed between groups (Fig. 1, Supplementary Table S1).

The only CBC measurements that differed significantly between groups were Ht (F_1,18_ = 6.81, *p* = 0.018) and the monocyte count (F_1,18_ = 5.55, *p* = 0.030) (Table 1).

**Table 1:** Results of the RM-ANOVA on ranks investigating between-timepoints (within-subject) and between-group (between-subject) difference in complete blood count and blood chemistry measurements.

	Sphericity assumption		Time effect		Group effect		Time × Group effect	
	Mauchly’s test *p*-value	Epsilon^a^	F-statistic df_1_ = 7^b^ df_2_ = 126^b^	*p*-value	F-statistic df_1_ = 1 df_2_ = 18	*p*-value	F-statistic df_1_ = 7^b^ df_2_ = 126^b^	*p*-value
Complete blood count measurements								
RBC [T/L]	0.089	-	11.2	< 0.001*	2.11	0.164	4.20	< 0.001*
HGB [mmol/L]	0.765	-	10.6	< 0.001*	2.66	0.121	1.69	0.116
Ht [%]	0.495	-	8.18	< 0.001*	6.81	0.018*	5.86	< 0.001*
MCV [fl]	0.002*	0.57	5.47 (4.01, 72.1)	0.001*	1.26	0.277	2.16 (4.01, 72.1)	0.082
MCH [fmol]	0.044*	0.66	12.9 (4.60, 82.9)	< 0.001*	0.35	0.563	2.77 (4.60, 82.9)	0.026*
MCHC [mmol/L]	0.581	-	9.86	< 0.001*	0.32	0.582	6.71	< 0.001*
WBC [G/L]	0.573	-	2.93	0.007*	1.11	0.306	0.51	0.828
Heterophils [G/L]	0.833	-	2.15	0.043*	0.77	0.393	1.46	0.189
Lymphocytes [G/L]	0.360	-	3.67	0.001*	0.67	0.425	0.47	0.856
Monocytes [G/L]	0.906	-	3.58	0.002*	5.55	0.030^c^*	1.83	0.087
Eosinophils [G/L]	0.386	-	0.47	0.857	0.02	0.889	0.38	0.914
Basophils [G/L]^d^	-	-	-	-	-	-	-	-
Blood chemistry measurements								
Amylase [U/L]	< 0.001*	0.51	5.94 (3.57,64.2)	0.001*	3.22	0.090	1.38 (3.57,64.2)	0.218
ALP [U/L]	0.062	-	9.45	< 0.001*	0.48	0.499	0.89	0.521
AST [U/L]	0.182	-	3.33	0.003*	0.06	0.817	1.23	0.289
CK [U/L]	0.234	-	8.18	< 0.001*	0.01	0.939	0.98	0.451
GGT [U/L]	0.901	-	2.39	0.025*	3.86	0.065	1.01	0.430
LDH [U/L]	0.543	-	6.80	< 0.001*	0.05	0.827	0.53	0.808
Cholesterol [mmol/L]	0.208	-	7.54	< 0.001*	0.03	0.863	0.73	0.644
Total protein [g/dL]	0.167	-	13.8	< 0.001*	0.79	0.385	1.71	0.113
Bile acids [µmol/L]	0.190	-	4.72	< 0.001*	1.25	0.278	1.55	0.158
Uric acid [mg/dL]	0.234	-	5.18	< 0.001*	3.03	0.099	2.13	0.045*
Triglycerides [mmol/L]	0.890	-	10.2	< 0.001*	0.40	0.534	2.58	0.016*
Ca [mmol/L]	0.215	-	2.06	0.053	4.44	0.049*	0.59	0.764
K [mmol/L]	0.479	-	10.6	< 0.001*	0.19	0.671	2.81	0.010*
Na [mmol/L]	0.545	-	30.1	< 0.001*	3.41	0.081	0.62	0.740
P [mmol/L]	0.296	-	1.01	0.431	0.43	0.520	1.16	0.328

^a^ estimated according to Greenhouse-Geisser procedure.

^b^ or df_1_ = 7 × epsilon, df_2_ = 126 × epsilon, if the sphericity assumption violated.

^c^ no significant difference observed between groups in pairwise comparisons.

* significant at α = 0.05.

**Fig. 2:**
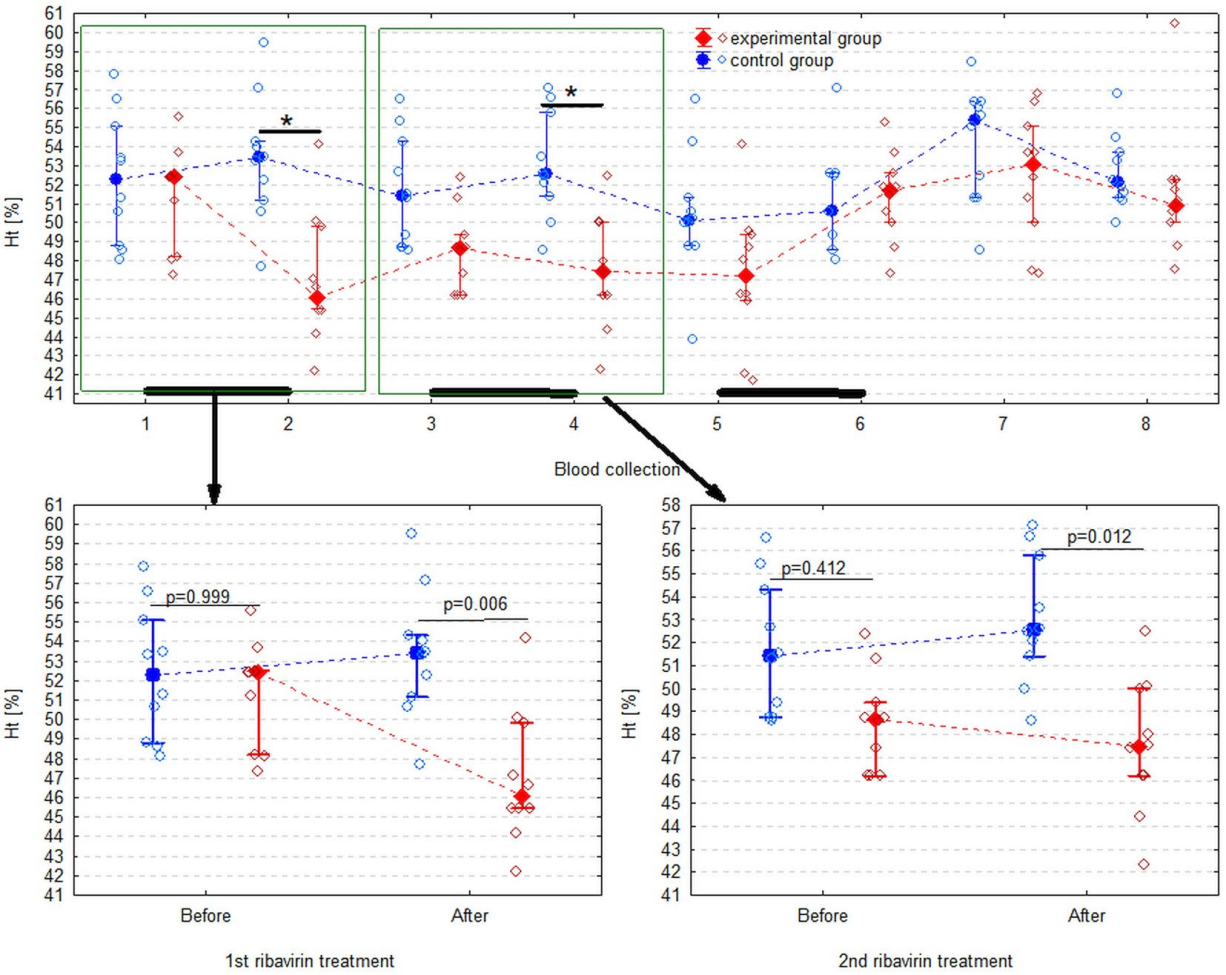
Hematocrit (packed cell volume, Ht) in the experimental and control group. It is presented as the median (solid dot or diamond), interquartile range (whiskers), and individual measurements (open dots or diamonds). Figure shows all 8 blood collections and separately the 1 st and 2nd ribavirin treatment. Asterisks (*) in the first graph indicate Ht significantly lower in the experimental group than in the control group according to the Tukey’s post-hoc test for equal groups. Black bars indicate periods of ribavirin treatment. The p-values in the 2nd and 3rd graph are from the Mann-Whitney U test without correction for multiple comparisons.

**Fig. 3:**
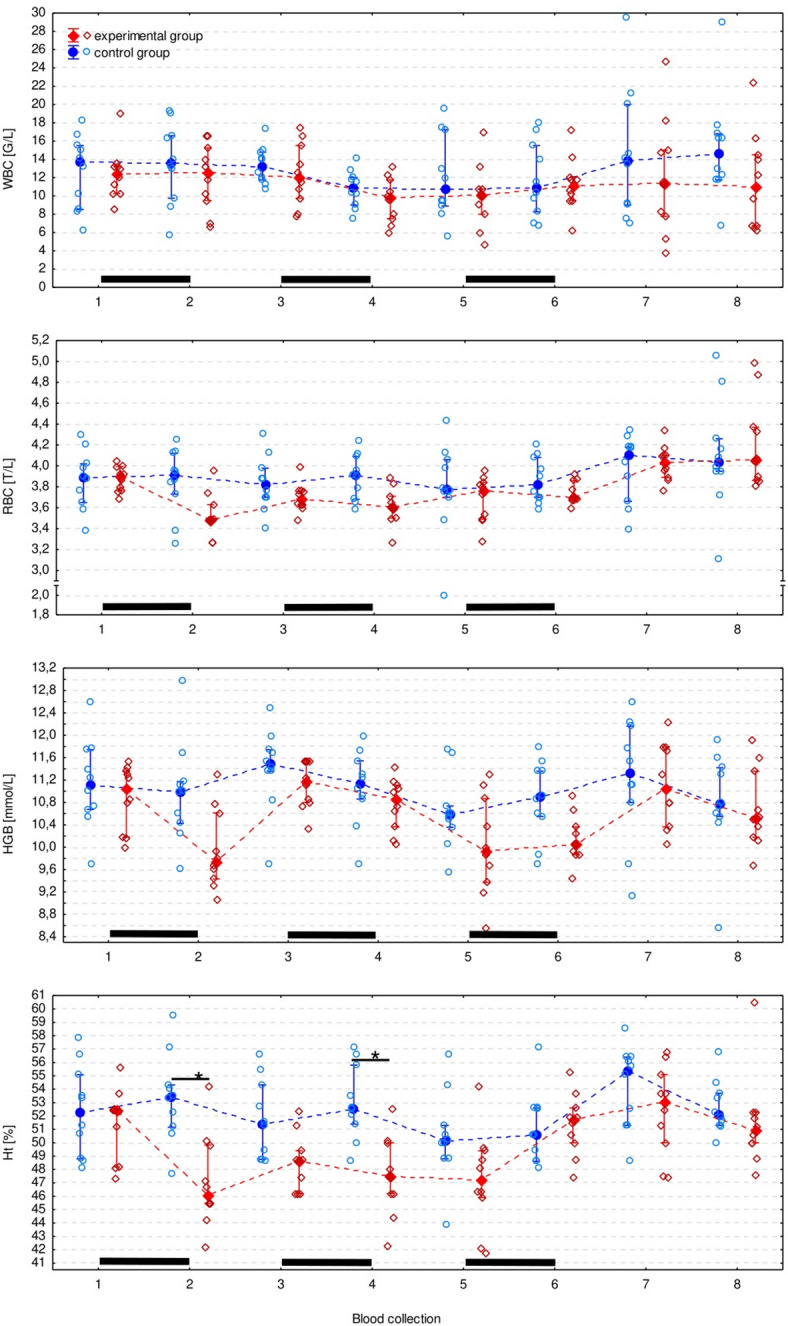
White blood cell count (WBC), red blood cell count (RBC), hemoglobin concentration (HGB), and hematocrit (packed cell volume, Ht) in experimental and control group presented as the median (solid dot or diamond). Asterisks (*) indicate Ht significantly lower in the experimental group than in the control group according to the Tukey’s post-hoc test for equal groups. Black bars indicate periods of ribavirin treatment.

**Fig. 4:**
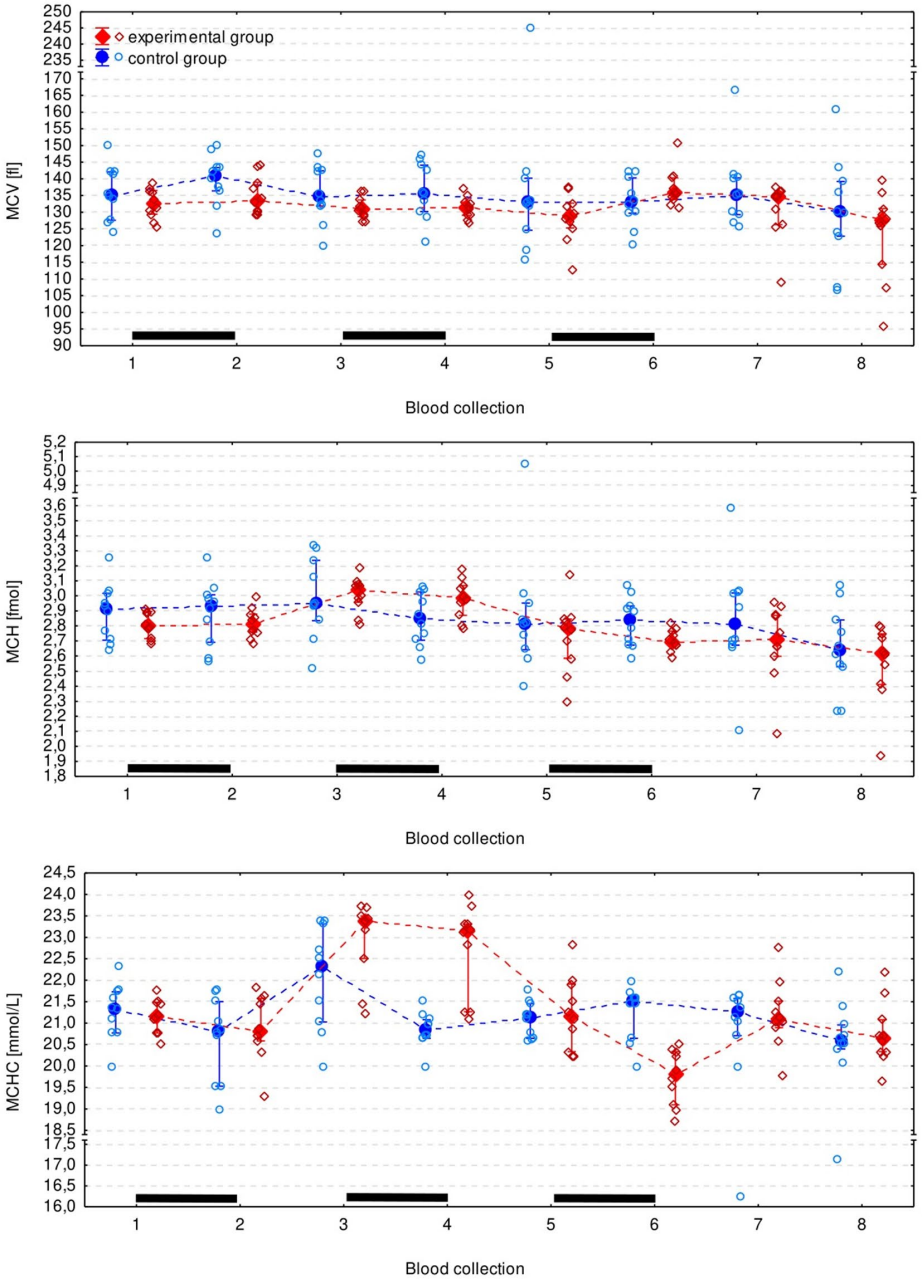
Erythrocyte (red blood cell) parameters in the experimental and control group presented as the median (solid dot or diamond), interquartile range (whiskers), and individual measurements (open dots or diamonds). Black bars indicate periods of ribavirin treatment.

**Fig. 5:**
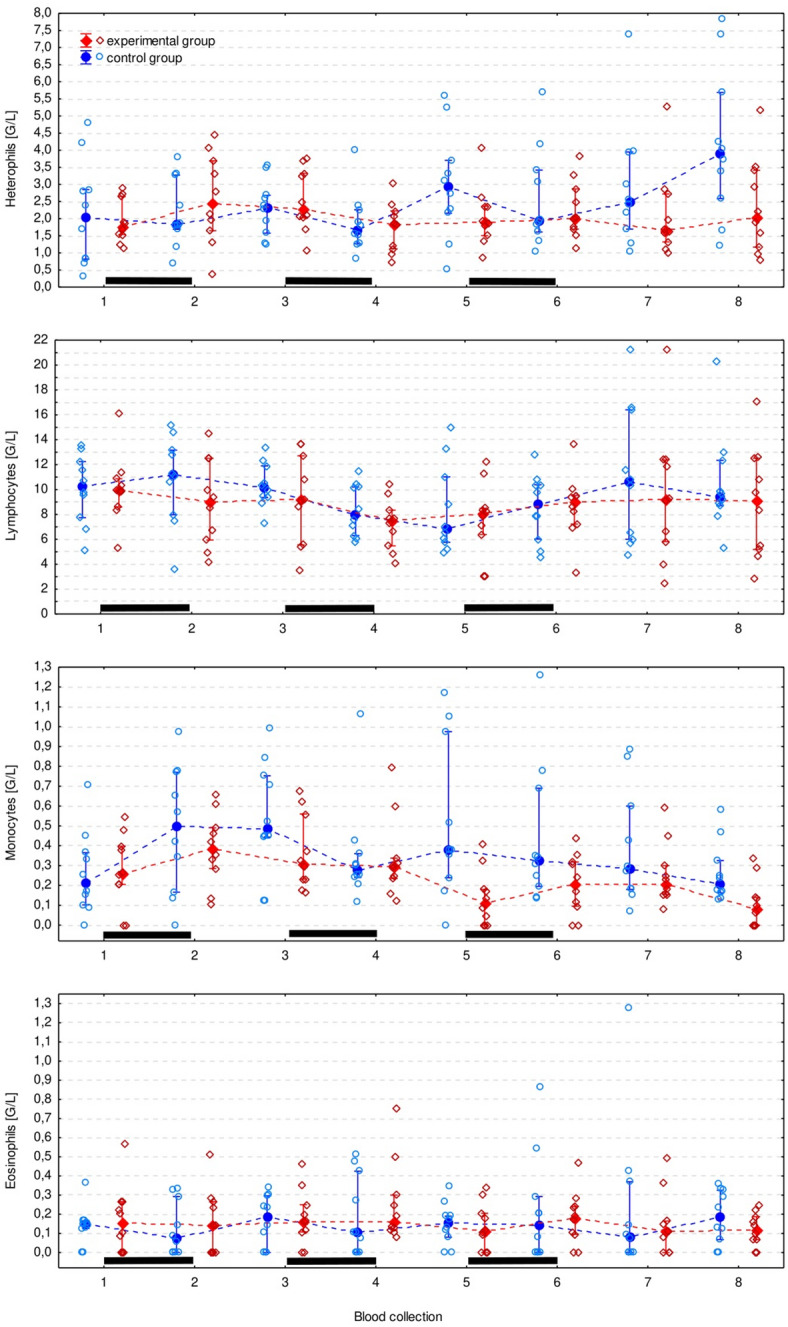
Elements of the differential leucogram in the experimental and control group. It has been presented as the median (solid dot or diamond), interquartile range (whiskers), and individual measurements (open dots or diamonds). Black bars indicate periods of ribavirin treatment.

Ht was significantly lower in the experimental than in control group after the 1 st (*p* = 0.006) and the 2nd course of ribavirin (*p* = 0.012) (Fig. 2). However, it was an isolated observation and no other RBC parameters differed significantly between groups (Figs. 3 and 4). The monocyte count appeared to be significantly lower in the experimental group, however the difference did not turn out to be significant in pairwise comparisons. All CBC measurements (except for the eosinophil count) significantly changed over time, but there was no regular pattern of the changes (Figs. 3, 4 and 5, Supplementary Table S2).

**Fig. 6:**
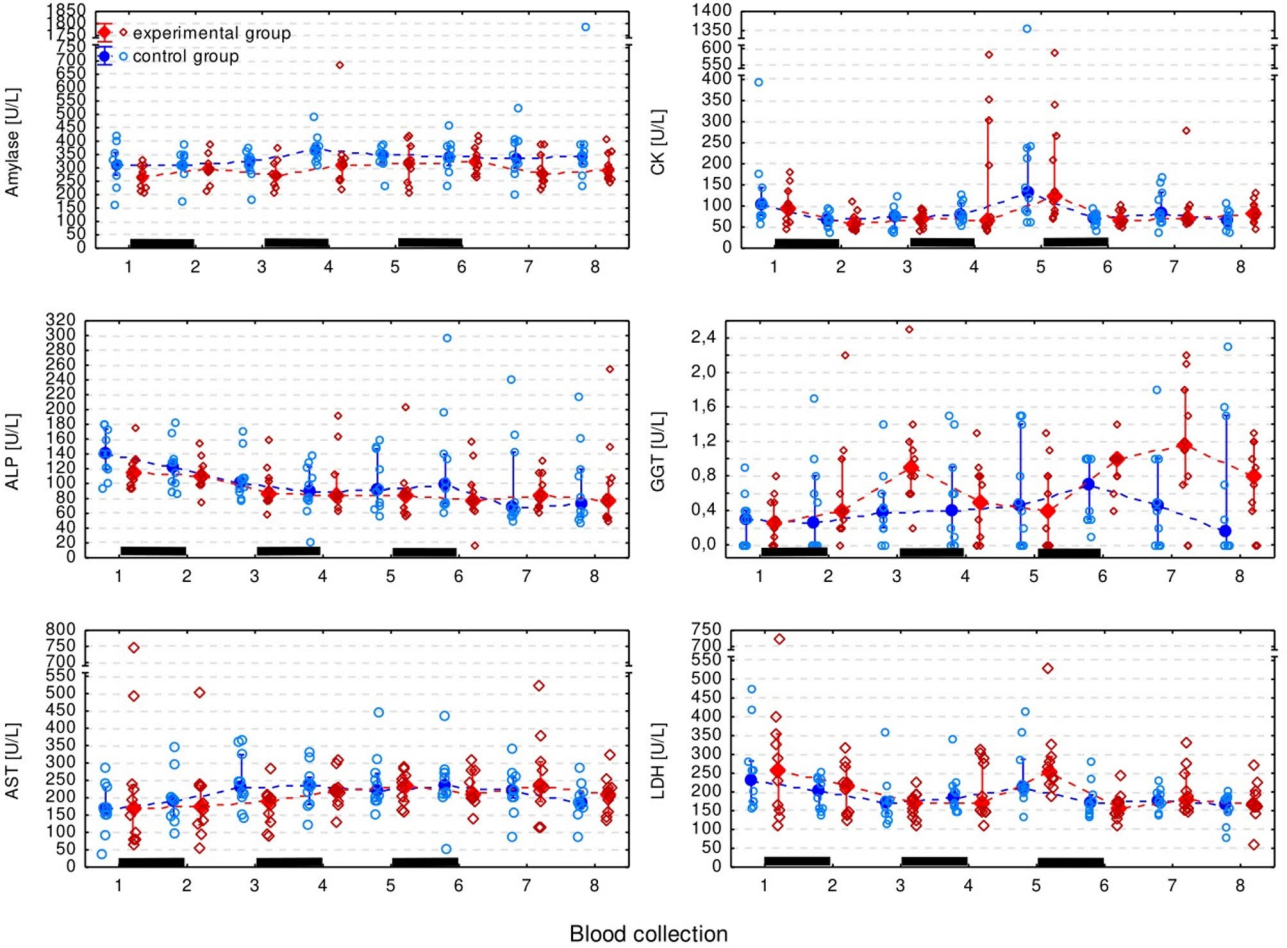
Plasma enzyme activity in the experimental and control group presented as the median (solid dot or diamond), interquartile range (whiskers), and individual measurements (open dots or diamonds). Black bars indicate periods of ribavirin treatment.

**Fig. 7:**
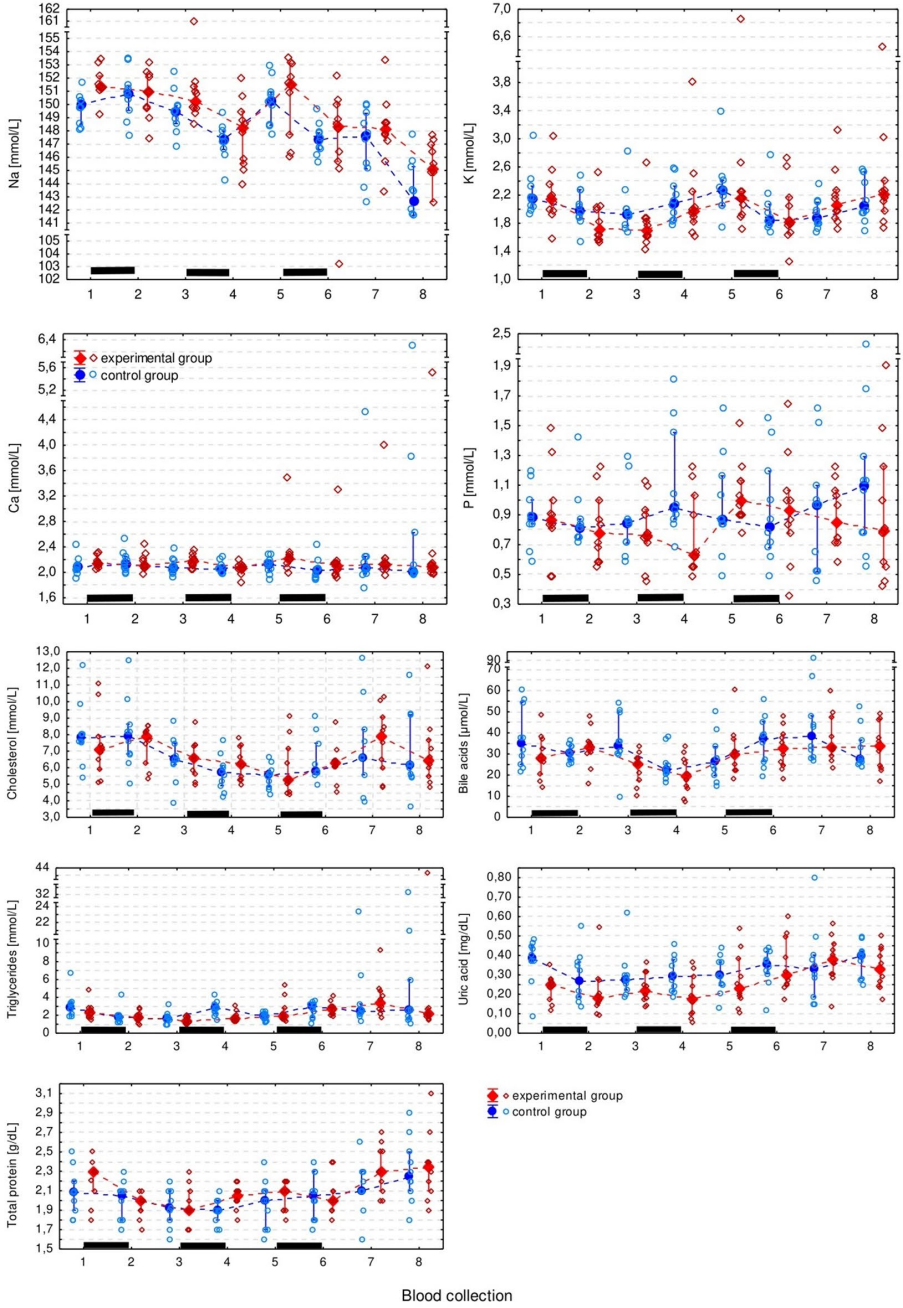
Plasma concentration of selected electrolytes and chemical compounds in the experimental and control group. It has been presented as the median (solid dot or diamond), interquartile range (whiskers), and individual measurements (open dots or diamonds). Black bars indicate periods of ribavirin treatment.

The only blood chemistry measurement that differed significantly between groups was Ca (F1,18 = 4.44, *p* = 0.049) which appeared to be higher in the experimental group. However, this was a borderline result which failed to prove significant in the pairwise comparisons. All blood chemistry measurements (except for the Ca and P) significantly changed over time, but there was no regular pattern of the changes (Figs. 6 and 7, Supplementary Table S2).

## Discussion

Our study showed that oral and intranasal administration of ribavirin at a total daily dose of 30 mg/kg BW in three 4-week-long therapeutic courses (12 weeks of treatment in total) did not result in any clinically apparent adverse reactions and clinically meaningful alterations of CBC and blood chemistry measurements.

There are only a few published studies on the use of ribavirin in birds^12,13,17^. Preliminary study on naturally infected grey parrots showed no measurable effect of ribavirin administered orally at dose 18 µg/kg BW on ABV shedding^13,23^. Hoppes et al. (2013) mentioned also that bad tolerance of higher doses by psittacine birds was observed (M. Rinder, unpublished results). Ribavirin dose used in our experiment is much higher (30 mg/kg BW) than 18 µg/kg BW but in our experiment influence of ribavirin therapy on bornaviral shedding was not studied. As no details are available regarding ribavirin doses which were badly tolerated, it is impossible to compare it with our results.

Due to the lack of studies focused on the safety of ribavirin in birds it is worth highlighting the results of studies conducted on other animals. As mentioned above, in rats, monkeys and human the major adverse effect of ribavirin treatment is haemolytic anemia. In human medicine it is a common cause of ribavirin dose reduction during therapy (in approximately 7–40% of patients)^24^. In connection with the above described mechanism of haemolytic anemia ribavirin has been shown to cause a significant decrease in RBC count, HGB concentration and Ht in rats, monkeys and humans which reaches its maximum in a few weeks, does not increase in severity with continued dosing and is reversible after cessation of ribavirin administration^25^. In one study, rats were given similar dose of ribavirin to that in our study (30 mg/kg/day for 5 days/week for 4 weeks). This treatment regime resulted in a significant decrease in absolute neutrophils count, RBC count, HGB, Ht and liver total iron binding capacity (TIBC)^26^. In our study the only hemathological measurement which differed significantly between groups during the study period was Ht while RBC and HGB concentration did not appear to be altered by ribavirin administration. The total iron binding capacity (TIBC) was not measured in our study. These differences could result from different pharmacokinetic and toxicologic properties of ribavirin in different species. These conclusions are supported by similar dosage regimens in our study concerning cockatiels and in the study conducted by Abd-Elmonem (2018) on rats.

Similar observations of the decrease in hematological parameters have been made in two other studies which aimed to compare the safety of viramidine and ribavirin^21,27^. Doses of ribavirin used in these experiments were even higher than in our study (60, 90 and 120 mg/kg/day in rats, 300 mg/kg/day in monkeys). Dadgostari et al. (2004) tested not only hematological parameters but also serum chemistry: alanine aminotransferase (ALT), albumin, albumin/globulin ratio (calculated) (A/G), ALP, AST, Ca, chloride (Cl), total cholesterol, creatinine, glucose, phosphorus, potassium, sodium, total bilirubin, total protein, triglycerides, blood urea nitrogen. After 28 days of treatment with dose 120 mg/kg/day in rats differences in values of ALP (lower), cholesterol (lower), albumin (higher), total protein (higher) were noted. These changes resolved after 28-day recovery period. In contrast, lower dose (90 mg/kg BW once daily) administered to rats for 6 months did not result in any changes in values of tested blood chemistry parameters. Blood chemistry parameters tested in our study were similar which allowed us to evaluate the function of major organs such as kidneys and liver. However, differences included additional parameters tested in our study (bile acids, uric acid, GGT, LDH) but also the lack of some parameters tested by Dadgostari et al. (2004) in our study (albumin, albumin/globulin ratio, creatinine, globulin, glucose, total bilirubin, blood urea nitrogen). However, except albumin concentration, all parameters which differed in rats after ribavirin treatment at dose 120 mg/kg/day were also tested in our study. Lower dose used in our study (30 mg/kg BW) did not result in changes in biochemical parameters similar to these observed in the rats that received ribavirin at dose 120 mg/kg. Similarly, when ribavirin was given half-by-half orally and intranasally no alterations in tested parameters were observed. It is worth mentioning that despite the presence of choana through which part of the drug given intranasally enters oral cavity^28^, the substance has still a chance to be absorbed by nasal mucosa similarly to mammals^29^ which makes it potentially useful administration route when pathologic process is located in central nervous system (CNS) such as in ABV infection^5^. It has been described that in mammals oral administration route could result in poor CNS penetration because of the presence of blood-brain barrier^30^. Oral administration route usually results in insufficient blood-brain barrier penetration. To increase the bioavailability of ribavirin in the CNS, scientists look for alternative administration routes^31^ or innovative formulations^32,33^. Intranasal administration route is one of the solutions considered owing to many advantages such as: non-invasiveness, accessibility, ease of administration and patient compliance^29^. In mammals, due to the presence of the olfactory receptor neurons, the olfactory region is the only site where the CNS is in its own way in contact with the external environment which makes it potentially easier to reach the cerebrospinal fluid when drug is administered into the nasal cavity^29^. Moreover, in dogs and rats the olfactory mucosa represents respectively 77% and 50% of the nasal mucosal surface^34^. Studies confirmed the positive outcome of nose-to-brain delivery for drug molecules^35,36^ and, even, for living cells^37,38^. Despite the historical assumptions that birds have very weak or no sense of smell^39,40^ and the fact that there is little data about size of olfactory epithelium compared to the nasal mucosal surface in avian species^41–43^, currently it is known that morphology of the olfactory systems in birds are similar to other vertebrates^44^, e.g. olfactory bulb size in birds varies between species depending on bird and brain size, phylogeny and behavior parallelly to that of other vertebrates^45^. Even though the blood-brain barrier in birds has lesser complexity than in mammals, it still plays an important role^46^. However, further studies in avian species to assess pharmacokinetic of ribavirin when administered intranasally are warranted.

In human medicine, ribavirin is used orally almost exclusively in combination with IFN-α which makes it difficult to define side the effects attributable only to ribavirin^47–49^. However, amongst adverse effects of HCV infection therapy with ribavirin combined with Peg-IFN-α anemia, neutropenia, thrombocytopenia, skin conditions, moderate to severe depression, colitis, pancreatitis, elevated serum liver enzymes, autoimmune hepatitis, thyrotoxicosis, flare of autoimmune conditions, sarcoidosis, interstitial pneumonitis, ophthalmologic conditions have been described^47,49^. When ribavirin was used alone for treatment of HCV infection, the most commonly reported adverse events were infection, asthenia, abdominal pain, headache, cough, depression, reduction in HGB concentration with increase of reticulocyte counts and bilirubin values^50^. Some of these symptoms such as abdominal pain or headache remain unknown in studies on parrots. However, daily clinical observations did not reveal any signs of distress, discomfort or pain in cockatiels treated with ribavirin. Plasma liver enzymes and HGB concentration values did not change significantly during experiment. Among tested CBC and blood chemistry parameters only decreased Ht corresponded with the changes described previously in human, monkeys and rats what has been mentioned above.

Our findings also align with broader toxicological research in birds, where hematological and biochemical alterations are frequently used as early markers of adverse effects. For example, aflatoxin B1 exposure in chickens induces significant hematobiochemical and histopathological changes, although protective strategies such as vitamin E and *Moringa oleifera* supplementation can mitigate these effects^51^. This underscores the value of systematic laboratory monitoring in evaluating both drug safety and potential interventions in avian species.

Direct extrapolation of pharmacokinetic and toxicologic properties of ribavirin from mammals to avian species is complicated. The results of our study are not sufficient to claim that ribavirin is safe for parrots due to some important limitations which are: lack of published pharmacokinetic studies, lack of data regarding ribavirin efficacy against avian viral diseases in vivo, only one dose used in the experiment. It is advisable to cover the above aspects during future research regarding avian viral diseases, especially caused by ABV and NDV.

## Conclusions

BW was changing significantly with time but it was not correlated with ribavirin treatment. Only Ht value significantly differed in experimental group after first and second ribavirin therapy when ribavirin was administered only to the crop. However, this difference was quite small and values still were within reference range (42–60%)^52^. Moreover, RBC and HGB did not differ significantly in analogous measurements. After third therapy when ribavirin was given half-by-half to the crop and intranasally this effect on hematocrit was not observed.

To conclude, ribavirin administered orally and half-by-half orally and intranasally in dose 30 mg/kg BW appears to be safe in cockatiels (*Nymphicus hollandicus*). Its negative influence on Ht is mild in the proposed dosage scheme.

## Supplementary Information

Below is the link to the electronic supplementary material.

Supplementary Material 1

## Data Availability

The datasets generated during and/or analysed during the current study are available from the corresponding author on reasonable request.
