# Supplementary material for: Safety of ribavirin in cockatiels (Nymphicus hollandicus) – a preliminary study
Source: Sci Rep. 2025 Nov 3;15:38407. doi: 10.1038/s41598-025-22268-9 (PMC12583684; doi:10.1038/s41598-025-22268-9)
Supplement: Supplementary file 1 — Supplementary Material 1 [file 41598_2025_22268_MOESM1_ESM.docx]

Supplementary Information

Supplementary Table S1. Body weight [g] of parrots in consecutive timepoints presented as the arithmetic means ± standard deviation and range in parentheses

|  | Date | Overall (n=20) | Experimental group (n=10) | Control group (n=10) |
| --- | --- | --- | --- | --- |
| 0 | 25.08.2023 | 110 ± 12 (94 – 128) | 111 ± 11 (94 – 128) | 108 ± 13 (94 – 128) |
|  | **28.08.2023** | **1^st^ blood collection and the onset of 1^st^ therapy** | | |
| 1 | 01.09.2023 | 108 ± 12 (89 – 130) | 108 ± 12 (92 – 130) | 108 ± 12 (89 – 126) |
| 2 | 08.09.2023 | 108 ± 12 (86 – 126) | 108 ± 13 (91 – 126) | 107 ± 12 (86 – 124) |
| 3 | 15.09.2023 | 103 ± 11 (84 – 120) | 104 ± 12 (85 – 120) | 101 ± 11 (84 – 116) |
| 4 | 22.09.2023 | 105 ± 11 (87 – 121) | 107 ± 11 (90 – 121) | 104 ± 10 (87 – 118) |
|  | **25.09.2023** | **2^nd^ blood collection and the end of 1^st^ therapy** | | |
| 5 | 02.10.2023 | 100 ± 11 (77 – 120) | 100 ± 11 (85 – 120) | 99 ± 12 (77 – 118) |
| 6 | 06.10.2023 | 99 ± 11 (77 – 119) | 100 ± 11 (84 – 117) | 97 ± 12 (77 – 119) |
|  | **09.10.2023** | **3^rd^ blood collection and the onset of 2^nd^ therapy** | | |
| 7 | 13.10.2023 | 96 ± 11 (73 – 115) | 97 ± 12 (75 – 111) | 95 ± 12 (73 – 115) |
| 8 | 20.10.2023 | 96 ± 10 (74 – 113) | 98 ± 9 (84 – 113) | 95 ± 12 (74 – 112) |
| 9 | 27.10.2023 | 96 ± 10 (75 – 113) | 98 ± 9 (86 – 113) | 94 ± 10 (75 – 108) |
| 10 | 03.11.2023 | 96 ± 9 (76 – 112) | 97 ± 10 (86 – 112) | 94 ± 9 (76 – 104) |
|  | **06.11.2023** | **4^th^ blood collection and the end of 2^nd^ therapy** | | |
| 11 | 10.11.2023 | 95 ± 10 (73 – 112) | 98 ± 10 (85 – 112) | 92 ± 9 (73 – 104) |
| 12 | 17.11.2023 | 93 ± 9 (74 – 114) | 96 ± 10 (83 – 114) | 91 ± 8 (74 – 101) |
|  | **20.11.2023** | **5^th^ blood collection and the onset of 3^rd^ therapy** | | |
| 13 | 24.11.2023 | 90 ± 9 (73 – 110) | 92 ± 10 (81 – 110) | 87 ± 8 (73 – 98) |
| 14 | 01.12.2023 | 89 ± 8 (74 – 106) | 92 ± 8 (81 – 106) | 87 ± 8 (74 – 99) |
| 15 | 08.12.2023 | 92 ± 8 (75 – 106) | 93 ± 9 (80 – 106) | 91 ± 8 (75 – 100) |
| 16 | 15.12.2023 | 92 ± 9 (75 – 107) | 92 ± 10 (78 – 107) | 91 ± 9 (75 – 100) |
|  | **18.12.2023** | **6^th^ blood collection and the end of 3^rd^ therapy** | | |
| 17 | 23.12.2023 | 94 ± 10 (76 – 115) | 96 ± 11 (81 – 115) | 93 ± 9 (76 – 104) |
| 18 | 29.12.2023 | 98 ± 12 (76 – 121) | 100 ± 12 (87 – 121) | 95 ± 11 (76 – 109) |
| 19 | 06.01.2024 | 101 ± 14 (76 – 131) | 104 ± 14 (93 – 131) | 99 ± 14 (76 – 117) |
| 20 | 12.01.2024 | 102 ± 14 (81 – 133) | 105 ± 15 (92 – 133) | 99 ± 14 (81 – 122) |
|  | **15.01.2023** | **7^th^ blood collection** |  |  |
| 21 | 19.01.2024 | 101 ± 14 (83 – 129) | 103 ± 14 (90 – 129) | 99 ± 14 (83 – 126) |
| 22 | 26.01.2024 | 102 ± 14 (86 – 132) | 103 ± 16 (86 – 132) | 101 ± 13 (86 – 126) |
| 23 | 02.02.2024 | 100 ± 14 (81 – 130) | 101 ± 15 (82 – 130) | 100 ± 15 (81 – 126) |
| 24 | 10.02.2024 | 100 ± 12 (84 – 126) | 101 ± 13 (84 – 126) | 100 ± 12 (85 – 123) |
| 25 | 16.02.2024 | 100 ± 12 (84 – 132) | 102 ± 14 (85 – 132) | 99 ± 11 (84 – 122) |
| 26 | 23.02.2024 | 100 ± 12 (83 – 129) | 102 ± 13 (87 – 129) | 99 ± 11 (83 – 123) |
| 27 | 03.03.2024 | 101 ± 13 (86 – 133) | 104 ± 13 (88 – 133) | 99 ± 12 (86 – 123) |
| 28 | 08.03.2024 | 100 ± 13 (83 – 135) | 103 ± 15 (87 – 135) | 97 ± 12 (83 – 123) |
|  | **11.03.2024** | **8^th^ blood collection** |  |  |
| 29 | 15.03.2024 | 100 ± 13 (83 – 132) | 102 ± 14 (88 – 132) | 97 ± 12 (83 – 121) |
| 30 | 22.03.2024 | 99 ± 11 (85 – 123) | 101 ± 11 (89 – 123) | 98 ± 11 (85 – 119) |

Supplementary Table S2. Results of the complete blood count and blood chemistry analysis in 8 consecutive time points presented as the median, interquartile range, and range in parentheses for all 20 parrots because no significant between-group differences were identified. The only exception was Ht, therefore values of cockatiels from each group are also presented.

| Blood collection | 1 | 2 | 3 | 4 | 5 | 6 | 7 | 8 |
| --- | --- | --- | --- | --- | --- | --- | --- | --- |
|  | 28.08.2023 | 25.09.2023 | 09.10.2023 | 06.11.2023 | 20.11.2023 | 18.12.2023 | 15.01.2024 | 11.03.2024 |
|  | 1^st^ course of ribavirin | | 2^nd^ course of ribavirin | | 3^rd^ course of ribavirin | |  |  |
| Complete blood count measurement | | | | | | | | |
| WBC [G/L] | 13.1, 10.2 – 14.6 (6.3 – 19.0) | 13.3, 9.6 – 16.4 (5.8 – 19.3) | 12.5, 11.0 – 14.9 (7.8 – 17.5) | 10.3, 8.3 – 11.9 (6.0 – 14.1) | 10.1, 8.4 – 13.1 (4.7 – 19.5) | 11.1, 9.5 – 14.1 (6.3 – 18) | 12.5, 8 – 16.6 (3.8 – 29.5) | 12.6, 8.3 – 16.3 (6.3 – 29.0) |
| RBC [T/L] | 3.9, 3.8 – 4.0 (3.4 – 4.3) | 3.7, 3.5 – 4.0 (3.3 – 4.3) | 3.7, 3.6 – 3.9 (3.4 – 4.3) | 3.7, 3.6 – 3.9 (3.3 – 4.2) | 3.8, 3.5 – 3.9 (2.0 – 4.4) | 3.7, 3.7 – 3.9 (3.6 – 4.2) | 4.1, 3.9 – 4.2 (3.4 – 4.3) | 4.1, 3.9 – 4.4 (3.1 – 5.1) |
| HGB [mmol/L] | 11.1, 10.6 – 11.4 (9.7 – 12.6) | 10.5, 9.7 – 11.1 (9.1 – 13.0) | 11.4, 10.8 – 11.5 (9.7 – 12.5) | 11.0, 10.5 – 11.3 (9.7 – 12.0) | 10.4, 9.8 – 10.8 (8.6 – 11.7) | 10.5, 9.9 – 10.9 (9.4 – 11.8) | 11.2, 10.6 – 11.8 (9.1 – 12.6) | 10.6, 10.4 – 11.4 (8.6 – 11.9) |
| Ht [%] | 52, 49 – 54 (47 – 58) | 50, 46 – 54 (42 – 60) | 49, 49 – 52 (46 – 57) | 50, 47 – 53 (42 – 57) | 49, 46 – 50 (42 – 57) | 51, 49 – 53 (47 – 57) | 54, 51 – 56 (47 – 59) | 52, 50 – 53 (48 – 61) |
| Experimental | 52, 48 – 53  (47 – 56) | 46, 45 – 50  (42 – 54) * | 49, 46 – 49  (46 – 52) | 47, 46 – 50  (42 – 53) * | 47, 46 – 49  (42 – 54) | 52, 50 – 53  (47 – 55) | 53, 50 – 55  (47 – 57) | 51, 50 – 52  (48 – 61) |
| Control | 52, 49 – 55  (48 – 58) | 53, 51 – 54  (48 – 60) | 51, 49 – 54  (49 – 57) | 53, 51 – 56  (49 – 57) | 50, 49 – 51  (44 – 57) | 51, 49 – 53  (48 – 57) | 55, 51 – 56  (49 – 59) | 52, 51 – 54  (50 – 57) |
| Tukey’s test p-value | 0.999 | 0.006* | 0.412 | 0.012* | 0.738 | 0.999 | 0.999 | 0.991 |
| MCV [fl] | 134, 129 – 138 (124 – 150) | 137, 131 – 143 (124 – 150) | 132, 129 – 136 (120 – 147) | 133, 129 – 136 (121 – 147) | 132, 125 – 135 (113 – 245) | 135, 132 – 140 (120 – 151) | 135, 128 – 137 (109 – 166) | 129, 119 – 136 (96 – 161) |
| MCH [fmol] | 2.8, 2.7 – 2.9 (2.6 – 3.3) | 2.8, 2.7 – 3.0 (2.6 – 3.3) | 3.0, 2.9 – 3.1 (2.5 – 3.3) | 3, 2.8 – 3.0 (2.6 – 3.2) | 2.8, 2.6 – 2.9 (2.3 – 5.0) | 2.7, 2.7 – 2.9 (2.6 – 3.1) | 2.7, 2.7 – 2.9 (2.1 – 3.6) | 2.6, 2.5 – 2.8 (1.9 – 3.1) |
| MCHC [mmol/L] | 21.2, 20.8 – 21.5 (20.0 – 22.3) | 20.7, 20.4 – 21.5 (19.0 – 21.8) | 22.9, 21.5 – 23.4 (20.0 – 23.7) | 21.2, 20.8 – 23.2 (20.0 – 24.0) | 21.1, 20.6 – 21.5 (20.2 – 22.8) | 20.5, 19.8 – 21.5 (18.7 – 21.9) | 21.1, 20.8 – 21.5 (16.2 – 22.8) | 20.6, 20.3 – 21.0 (17.1 – 22.2) |
| Heterophils [G/L] | 1.8, 1.2 – 2.8 (0.31 – 4.8) | 2.1, 1.7 – 3.3 (0.38 – 4.5) | 2.3, 1.8 – 3.3 (1.1 – 3.8) | 1.8, 1.2 – 2.2 (0.72 – 4) | 2.3, 1.7 – 3.2 (0.5 – 5.6) | 2, 1.7 – 3.2 (1 – 5.7) | 2.1, 1.4 – 2.9 (1.0 – 7.4) | 3.2, 1.6 – 4.2 (0.81 – 7.8) |
| Lymphocytes [G/L] | 9.9, 8.5 – 12.0 (5.1 – 16) | 10.0, 7.1 – 13.0 (3.6 – 15) | 9.8, 8.8 – 12 (3.5 – 14.0) | 7.6, 6.2 – 9.9 (4.1 – 11.0) | 7.5, 5.9 – 9.9 (3.0 – 15.0) | 9.0, 7.1 – 10.0 (3.3 – 14.0) | 9.8, 5.9 – 12.0 (2.5 – 21.0) | 9.4, 6.7 – 12 (2.8 – 20.0) |
| Monocytes [G/L] | 0.25, 0.13 – 0.39 (0 – 0.71) | 0.42, 0.23 – 0.63 (0 – 0.98) | 0.45, 0.23 – 0.65 (0.12 – 0.99) | 0.29, 0.24 – 0.35 (0.12 – 1.10) | 0.21, 0.07 – 0.40 (0 – 1.20) | 0.28, 0.14 – 0.35 (0 – 1.30) | 0.24, 0.16 – 0.44 (0.07 – 0.89) | 0.15, 0.08 – 0.27 (0 – 0.58) |
| Eosinophils [G/L] | 0.15, 0.04 – 0.22 (0 – 0.57) | 0.11, 0 – 0.28 (0 – 0.51) | 0.16, 0.05 – 0.27 (0 – 0.47) | 0.13, 0.09 – 0.36 (0 – 0.75) | 0.13, 0.04 – 0.20 (0 – 0.35) | 0.18, 0 – 0.26 (0 – 0.86) | 0.10, 0 – 0.27 (0 – 1.30) | 0.14, 0.07 – 0.24 (0 – 0.36) |
| Basophils [G/L] | 0, 0 – 0 (0 – 0.16) | 0, 0 – 0 (0 – 0.13) | 0, 0 – 0 (0 – 0) | 0, 0 – 0 (0 – 0) | 0, 0 – 0 (0 – 0.2) | 0, 0 – 0 (0 – 0.16) | 0, 0 – 0 (0 – 0) | 0, 0 – 0 (0 – 0.16) |
| Number of parrots in which Basophils were found | 3 / 20 (15%) | 2 / 20 (10%) | 0 / 20 (0) | 0 / 20 (0) | 4 / 20 (20%) | 2 / 20 (10%) | 0 / 20 (0) | 2 / 20 (0) |
| Blood chemistry measurement | | | | | | | | |
| Amylase [U/L] | 296, 231 – 322 (163 – 422) | 308, 290 – 346 (173 – 391) | 301, 272 – 335 (181 – 377) | 336, 309 – 374 (223 – 683) | 328, 306 – 368 (204 – 418) | 336, 291 – 377 (235 – 461) | 309, 265 – 385 (199 – 522) | 317, 268 – 365 (231 – 1785) |
| ALP [U/L] | 125, 103 – 150 (92.6 – 180) | 113, 100 – 131 (75 – 181) | 95.7, 78.5 – 108 (58.5 – 170) | 85.1, 77.2 – 118 (20.3 – 193) | 87.3, 66.5 – 109 (56.4 – 203) | 84.3, 72.9 – 137 (16.5 – 298) | 72.2, 63.2 – 114 (49.1 – 240) | 78.3, 54.8 – 115 (47.7 – 254) |
| AST [U/L] | 164, 94.4 – 234 (36.4 – 748) | 186, 131 – 235 (57 – 503) | 202, 153 – 245 (89.6 – 366) | 220, 186 – 249 (119 – 330) | 232, 190 – 268 (150 – 445) | 218, 199 – 274 (52.2 – 435) | 225, 194 – 273 (86.2 – 525) | 200, 163 – 228 (85.8 – 326) |
| CK [U/L] | 102, 73.1 – 140 (46.2 – 393) | 64.3, 47.7 – 76.5 (39.1 – 111) | 72.8, 51 – 86.1 (39.2 – 122) | 75.4, 56.7 – 121 (43.4 – 582) | 127, 84.8 – 241 (61.1 – 1353) | 67.6, 58 – 86.2 (40 – 102) | 75, 63.3 – 108 (36.6 – 281) | 74.6, 57.6 – 96.9 (38.6 – 133) |
| GGT [U/L] | 0.3, 0 – 0.5 (0 – 0.9) | 0.4, 0 – 0.9 (0 – 2.2) | 0.6, 0.3 – 1.1 (0 – 2.5) | 0.5, 0 – 0.9 (0 – 1.5) | 0.5, 0 – 1.0 (0 – 1.5) | 1.0, 0.4 – 1.0 (0.1 – 1.4) | 0.8, 0.1 – 1.4 (0 – 2.2) | 0.6, 0 – 1.2 (0 – 2.3) |
| LDH [U/L] | 242, 163 – 340 (112 – 725) | 214, 148 – 238 (124 – 319) | 169, 140 – 187 (112 – 361) | 180, 148 – 252 (110 – 341) | 228, 208 – 291 (134 – 530) | 168, 142 – 183 (112 – 279) | 175, 158 – 205 (139 – 330) | 168, 147 – 192 (57.7 – 273) |
| Cholesterol [mmol/L] | 7.6, 6.3 – 8.0 (5.2 – 12.2) | 7.9, 6.6 – 8.4 (5.0 – 12.5) | 6.5, 5.8 – 7.4 (3.9 – 8.9) | 5.9, 5.2 – 6.8 (4.3 – 7.8) | 5.4, 4.8 – 6.3 (4.4 – 9.1) | 6.1, 5.7 – 6.8 (4.6 – 9.1) | 7.1, 5.8 – 8.8 (4.0 – 12.6) | 6.2, 5.5 – 8.0 (3.7 – 12.1) |
| Total protein [g/dL] | 2.2, 2.0 – 2.3 (1.8 – 2.5) | 2.0, 1.8 – 2.1 (1.7 – 2.3) | 1.9, 1.9 – 2.1 (1.6 – 2.3) | 2.0, 1.9 – 2.1 (1.7 – 2.2) | 2.1, 1.9 – 2.2 (1.6 – 2.4) | 2.0, 1.9 – 2.2 (1.7 – 2.4) | 2.2, 2.1 – 2.4 (1.6 – 2.7) | 2.3, 2.1 – 2.5 (1.8 – 3.1) |
| Bile acids [μmol/L] | 30.7, 24.2 – 38.1 (14.4 – 60.6) | 31.8, 27.4 – 35.1 (15.9 – 48.2) | 30.1, 23.2 – 34.9 (9.8 – 54.4) | 21.4, 17.1 – 26.5 (7.40 – 38.4) | 28.0, 22.2 – 35.3 (15.1 – 60.5) | 35.6, 25.6 – 42.3 (18.4 – 55.9) | 33.4, 28.9 – 47.9 (23.4 – 91.6) | 29.6, 24.8 – 41.8 (17.1 – 52.5) |
| Uric acid [mg/dL] | 0.28, 0.24 – 0.39 (0.09 – 0.49) | 0.20, 0.16 – 0.34 (0.10 – 0.55) | 0.25, 0.19 – 0.31 (0.14 – 0.62) | 0.24, 0.17 – 0.34 (0.05 – 0.46) | 0.27, 0.20 – 0.38 (0.11 – 0.54) | 0.333, 0.258 – 0.423 (0.12 – 0.6) | 0.35, 0.29 – 0.44 (0.14 – 0.80) | 0.38, 0.27 – 0.43 (0.17 – 0.50) |
| Triglycerides [mmol/L] | 2.66, 1.9 – 3.15 (1.46 – 6.76) | 1.82, 1.47 – 2.05 (0.963 – 4.35) | 1.54, 1.17 – 1.84 (0.927 – 3.29) | 2.12, 1.52 – 2.85 (1.30 – 4.32) | 1.94, 1.53 – 2.26 (1.17 – 5.45) | 2.79, 2.07 – 3.22 (1.1 – 4.21) | 2.72, 2.17 – 4.44 (1.3 – 23.4) | 2.17, 1.71 – 2.74 (1.08 – 43.4) |
| Ca [mmol/L] | 2.10, 2.06 – 2.19 (1.90 – 2.43) | 2.11, 2.08 – 2.24 (1.98 – 2.53) | 2.11, 2.05 – 2.20 (1.93 – 2.38) | 2.06, 1.99 – 2.10 (1.85 – 2.25) | 2.16, 2.06 – 2.28 (1.88 – 3.50) | 2.05, 1.95 – 2.15 (1.88 – 3.30) | 2.13, 2.04 – 2.23 (1.75 – 4.53) | 2.06, 2.00 – 2.23 (1.95 – 6.30) |
| K [mmol/L] | 2.15, 1.98 – 2.34 (1.59 – 3.05) | 1.92, 1.64 – 2.06 (1.54 – 2.53) | 1.84, 1.65 – 1.96 (1.43 – 2.82) | 2.01, 1.83 – 2.29 (1.61 – 3.81) | 2.22, 2.05 – 2.36 (1.72 – 6.86) | 1.83, 1.75 – 2.12 (1.25 – 2.77) | 1.97, 1.8 – 2.18 (1.66 – 3.12) | 2.15, 1.95 – 2.47 (1.69 – 6.45) |
| Na [mmol/L] | 151, 150 – 151 (148 – 153) | 151, 150 – 152 (147 – 154) | 150, 149 – 151 (147 – 161) | 148, 146 – 149 (144 – 152) | 151, 148 – 152 (146 – 154) | 148, 146 – 149 (103 – 152) | 148, 146 – 149 (143 – 153) | 144, 142 – 146 (139 – 148) |
| P [mmol/L] | 0.88, 0.82 – 1.00 (0.49 – 1.49) | 0.81, 0.71 – 0.94 (0.55 – 1.42) | 0.81, 0.71 – 0.92 (0.45 – 1.29) | 0.90, 0.63 – 1.10 (0.49 – 1.81) | 0.94, 0.86 – 1.15 (0.49 – 1.62) | 0.87, 0.70 – 1.13 (0.36 – 1.65) | 0.97, 0.68 – 1.08 (0.45 – 1.62) | 0.90, 0.60 – 1.26 (0.42 – 2.42) |

* significant at α=0.05
